# Target Recognition Based on Millimeter-Wave-Sensed Point Cloud Using PointNet++ Model

**DOI:** 10.3390/s25185694

**Published:** 2025-09-12

**Authors:** Xianxian He, Haiyu Ding, Rongyan Xi, Jing Dong, Jing Jin, Qixing Wang, Chunju Shao, Xiao Dong, Yunhua Zhang

**Affiliations:** 1 Future Research Laboratory, China Mobile Research Institute, Beijing 100053, China; hexianxian@chinamobile.com (X.H.);; 2 CAS Key Laboratory of Microwave Remote Sensing, National Space Science Center, Chinese Academy of Sciences, Beijing 100190, China; dongxiao@mirslab.cn; 3 School of Electronic, Electrical, and Communication Engineering, University of Chinese Academy of Sciences, Beijing 100049, China

**Keywords:** gait recognition, millimeter-wave radar point cloud, extracted limb motion data, PointNet++ model

## Abstract

During walking, the human lower limbs primarily support the body and drive forward motion, while the arms exhibit greater variability and flexibility without bearing such loads. In gait-based target recognition, collecting exhaustive arm-motion data for training is challenging, and unseen arm movements during testing may degrade the performance. This paper investigates the impact of arm movements on radar-based gait recognition and proposes a gait recognition method using extracted lower limb motion data to mitigate interference from different arm motions. Gait data is collected via a millimeter-wave radar sensor encompassing four kinds of common arm movements, including natural arm swings, object-holding states, and irregular arm motions, from 11 volunteers. Using extracted lower limb motion data, millimeter wave point-cloud gait datasets covering diverse arm motions are generated. Three gait recognition experiments are conducted for comparing the performances of our proposed method using only lower limb data and existing method using all limb data, both based the on PointNet++ model. And the experimental results show that our method consistently outperforms existing methods, with a 22.9-percent improvement in accuracy. Results also show that the proposed method can enhance feature extraction, accelerate convergence, and achieve higher accuracy, especially with limited samples, and the highest recognition accuracy reaches 96.9%. In addition, in unseen arm movement cases, our method significantly outperforms existing methods, demonstrating superior robustness and recognition accuracy.

## 1. Introduction

With the rapid advancement of artificial intelligence and biometric technologies [1,2], gait recognition, as a non-contact identity authentication method, has gained extensive attention from both academia and industry in recent years [3,4]. This technology achieves accurate individual identification by analyzing unique gait characteristics during walking, such as stride length, walking speed, and body swing patterns [5,6,7]. Among various sensor technologies, radar stands out due to its distinctive advantages, including high robustness and all-weather applicability [8,9]. It not only can penetrate obstacles to capture dynamic human motion information but also can enable long-distance and continuous data acquisition without infringing personal privacy, through which immense application potential in gait recognition has been demonstrated [10,11,12].

When radar detects pedestrians, the received echo data encompasses motion signals from all limbs, including both lower limb movements and arm swings [13,14]. However, societal behavioral patterns have undergone significant changes with the proliferation of mobile internet [15]. For instance, walking while using smartphones has become ubiquitous [16], altering traditional arm swing patterns—such as reduced arm swings when holding a phone with one hand or near-static arm postures during two-handed texting. Additionally, gestural communication in social contexts further diversifies arm motion patterns [17]. These variations render arm movements more flexible and unpredictable compared to lower limb motions, imposing higher demands on the robustness and accuracy of gait recognition systems [18].

Existing gait recognition models often fail to encompass all potential arm motion states during training [19,20], leading to performance degradation when confronted with gait data featuring arm movements divergent from training samples. Current radar-based gait recognition research and publicly available datasets largely neglect the impact of diverse arm motions [21,22]. Therefore, investigating the influence of different arm movement patterns on gait recognition is crucial for enhancing the practicality of this technology.

In this paper, we propose a novel gait recognition method based on extracted lower limb motion data using a point-cloud deep network. The limb motions of a walker are captured by a millimeter-wave radar, and the lower limb motion point cloud is then extracted using the approach presented in [23], where an interferometric radar was used to separate and extract limb motion data. In this study, we collected gait data under varying arm motion states and constructed the corresponding gait dataset. After conducting comprehensive gait recognition experiments, we then analyze the effects of different arm movements on recognition performance. Experimental results have validated the effectiveness of the proposed method with significant improvements in recognition accuracy demonstrated for gait data with diverse arm motions.

The remainder of this paper is arranged as follows. Section 2 briefly presents the extraction of point-cloud data of human limbs. Based on the extracted lower limb data, the method for gait recognition and point-cloud network are developed in Section 3. Practical experiments are conducted with the results analyzed in Section 4, and finally the paper is concluded in Section 5.

## 2. Extracted Millimeter-Wave Point-Cloud Data of Human Limbs

When a human target is walking, multiple moving limbs typically exhibit simultaneous activities. The radar receives echoes aggregated from all these moving body parts, with their motion-related data superimposed in the received signal. To better analyze and identify human motion states, it is essential to separate and extract the data corresponding to individual moving limbs for targeted processing and interpretation.

**Figure 1:**
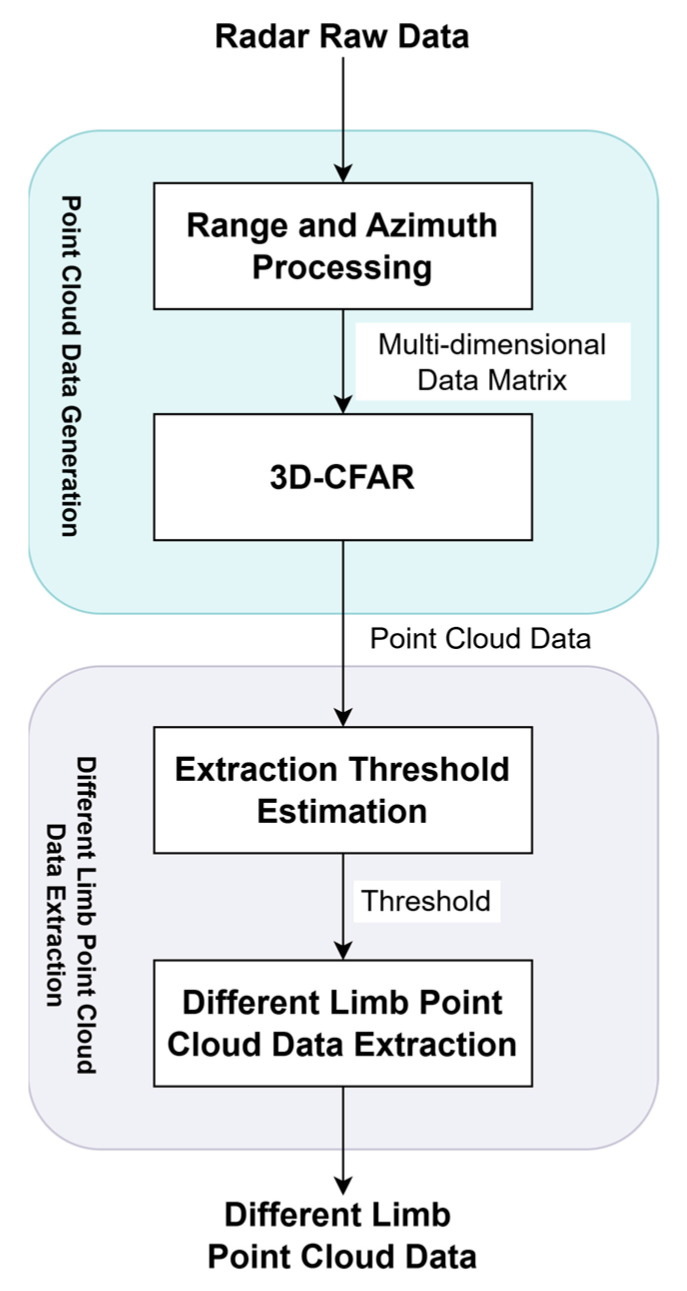
Extraction flow chart of different limb point-cloud data based on multi-channel radar.

In the application of gait recognition, the diverse motion states of arms can negatively impact recognition accuracy. To address this issue, we developed a method to separate and extract different limb motion data, as introduced in our previous work [23]. The processing flow for obtaining human motion point-cloud data and extracting data on different limbs consists of two main modules. The first module involves generating point-cloud data by performing pulse compression in the range direction and short-time Fourier transform in the azimuth direction on the raw radar echo data, resulting in a multi-dimensional data matrix encompassing range, velocity, and time information. Subsequently, 3D-CFAR (Constant False Alarm Rate) target detection is applied to this matrix to extract human motion point-cloud data. The second module focuses on separating different limb motion data. Based on the elevation threshold estimation method [23], the point cloud is divided into two components: the data above the threshold (representing arm motion) and the data below the threshold (representing lower limb motion). The extracted lower limb (leg) motion data is then utilized for gait-based identity recognition after mitigating the interference caused by arm movements. The overall workflow is illustrated in Figure 1.

Therefore, the point-cloud data of different moving limbs can be obtained by processing the radar raw data of human motion, as shown in Figure 1, and subsequent applications such as human gait recognition can be carried out.

## 3. Gait Recognition Based on Extracted Lower Limb Data Using PointNet++

### 3.1. Gait Recognition Based on Extracted Lower Limb (Leg) Data

Gait recognition is a biometric technology that identifies identity information based on the gait characteristics of a human whilst walking. Gait characteristics mainly include features such as the step frequency, step length, and walking posture of the human target, which are primarily contained in the motion information of legs; thus, it is possible to effectively extract gait features by analyzing the leg motion data.

**Figure 2:**
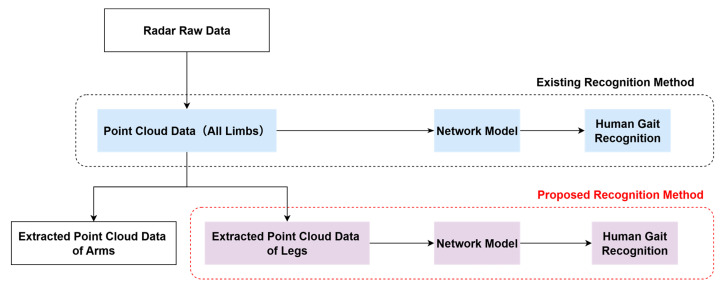
Proposed method of gait recognition using leg data only and existing method using all limb data.

To improve recognition accuracy, we propose a gait recognition method using leg motion data. As illustrated in Figure 2, the process begins by separating the full-body point cloud into arm and leg components using the previously developed extraction method. By utilizing only the leg point-cloud data, this method eliminates interference from arm movements, ensuring more reliable gait recognition.

**Figure 3:**
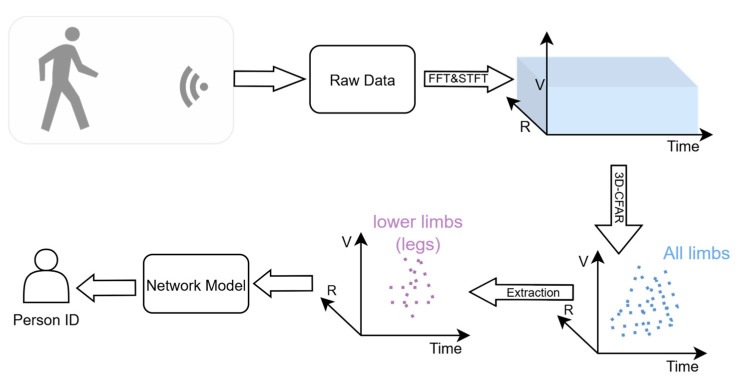
The pipeline of the proposed gait recognition method.

As shown in Figure 3, the proposed method is based on the point-cloud data of the legs to extract gait features and achieve human identity recognition. Firstly, interferometric radar or MIMO radar (multi-channel radar) is used to obtain the raw echo data of human movements. Here, the multi-channel radar configuration is used to obtain the angle information of targets, which can be used for the subsequent extraction of leg data. Then, the raw echo data is processed to obtain the range, velocity and time information of the target; that is, the three-dimensional (3D) cube data can be obtained. Then, target detection processing is carried out on the 3D cube to obtain the point cloud. It should be noted that, unlike the conventional point-cloud construction method, the point cloud in this paper is constructed in three dimensions: range, velocity and time. This construction method has been verified to be more effective in extracting target features based on millimeter-wave point clouds [24].

Existing gait recognition methods utilize point-cloud data of all limbs. However, the network model cannot differentiate which parts of the point cloud come from the legs and which come from the arms. Due to the diversity of arm movements, training samples must incorporate as many arm actions as possible, making data collection challenging and increasing training costs. To solve this, the point cloud of all the moving limbs is further processed, as shown in Figure 3. Using elevation angle data, we estimate a threshold and extract points below it to isolate leg the point cloud [23]. The model is then trained and tested exclusively on the leg point cloud for gait recognition.

### 3.2. Gait Recognition Using PointNet++ Model

Point-cloud data generally consists of coordinate points in three or more dimensions. In fields such as computer vision, robotics, and autonomous driving, it is often used to describe the surface of an object or a scene, playing a crucial role in practical applications. However, due to challenges such as disorderliness, sparsity, and irregularity in point-cloud data, directly applying well-established convolutional neural networks (CNNs) to raw point clouds remains difficult. Common preprocessing methods, such as projecting a point cloud onto 2D planes or voxelization [25], often lead to information loss.

**Figure 4:**
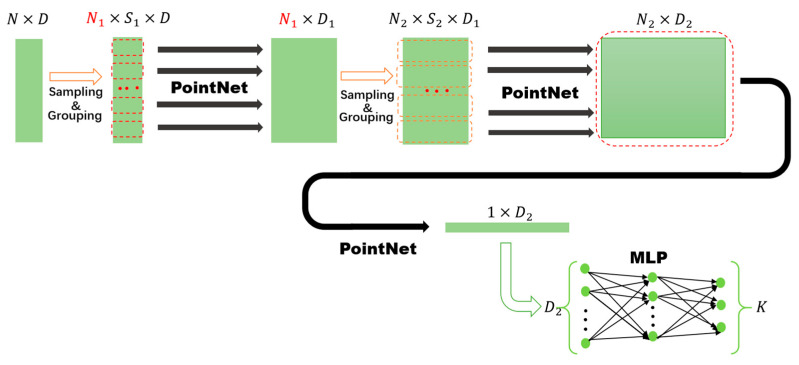
K-classification network model based on PointNet++.

**Figure 5:**
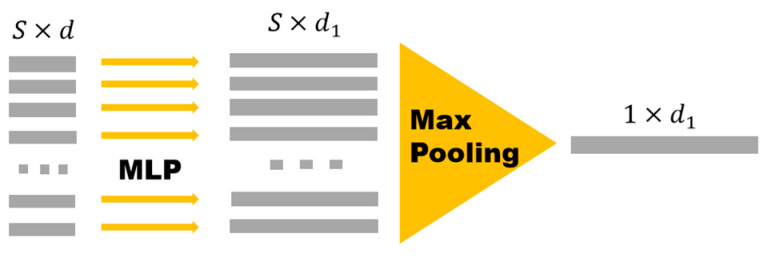
PointNet network structure.

PointNet++ [26] is shown in Figure 4 for point-cloud data containing N points, and each of them has D dimensions. The PointNet++ network model first samples the N points to obtain $N_{1}$ core points. Then, for each core point, $S_{1}$ neighboring points are grouped together, resulting in $N_{1}$ groups; i.e., each group contains $S_{1}$ points. Next, a PointNet [27] feature extraction (as shown in Figure 5) is performed on the $S_{1}$ points of each group, to obtain the data of $N_{1}\times D_{1}$ dimensions. By repeating the above operations, the local features of the point cloud can be hierarchically extracted. Figure 4 shows a network structure with two levels of hierarchical feature extraction. Finally, a feature vector of dimension $D_{2}$ can be obtained, which contains both local and global feature information of the point cloud. Therefore, by performing MLP processing on the feature vector, the classification and recognition of K categories can be achieved.

Figure 5 shows the basic structure of the PointNet network model. Its core idea is to directly process point clouds by introducing symmetric functions to solve the problem of the unordered nature of point clouds. In Figure 5, the point-cloud data consists of S points, with each point having d  dimensions. First, each point is processed by a Multilayer Perception (MLP) and then point-cloud data with $d_{1}$ dimensions is obtained. Subsequently, Max Pooling is used to implement the symmetric function so as to complete the feature extraction of the point cloud and obtain a feature vector of $d_{1}$ dimensions.

As can be seen from Figure 4, PointNet++ draws on the idea of hierarchical local feature extraction in convolutional network models to hierarchically extract local features and is based on the PointNet network model to directly extract features from point-cloud data.

In general, PointNet ++ is used for static point clouds. For dynamic point-cloud data, specialized network modules (such as long short-term memory networks) are required to extract temporal features. As we all know, human movements are time-varying. In this paper, by adopting a three-dimensional (range, velocity and time) point-cloud construction method, the temporal information is taken as one dimension constituting the representation of the point cloud; thus, the temporal features are integrated into the representation of the point cloud [24]. By using this point-cloud construction method, the point cloud of human movements can be regarded as a static point cloud, as shown in Figure 3. That is to say, gait recognition based on millimeter-wave point clouds can be completed only by using the PointNet++ model with range, velocity and time.

**Figure 6:**
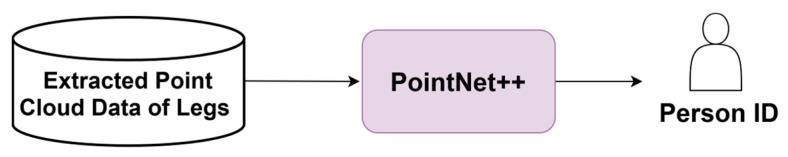
Gait recognition using PointNet ++ based on extracted leg point-cloud data.

In the experiments here, the point-cloud data of the extracted legs are used to recognize different gaits. That is, the walking data are collected and processed, and the extracted leg motion data is used to identify the human targets. The block diagram of the proposed gait recognition method is presented in Figure 6.

## 4. Gait Recognition Experiments and Results

### 4.1. Data Prepossessing

During the experiment, an L-shaped three-channel interferometric radar was employed to collect walking motion data from eleven volunteers, with particular attention to four distinct arm-movement scenarios: natural arm swinging (both arms swinging periodically), single-arm swinging (holding an object in one hand), no arm swinging (holding objects in both hands), and free arm swinging (unrestricted, non-rhythmic movements).

**Figure 7:**
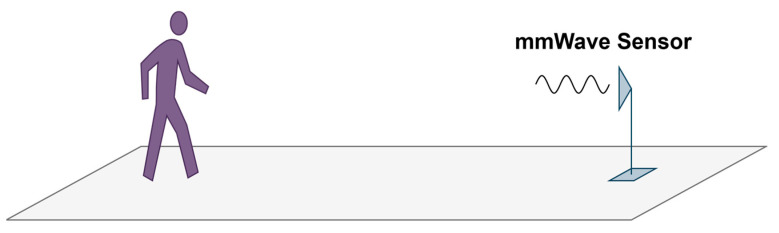
Gait data acquisition scenario based on multi-channel radar.

As shown in Figure 7, the gait data acquisition scenario is illustrated. The millimeter-wave radar sensor (mmWave sensor) is Texas Instruments’ AWR1843 in the band of 77 to 81 GHz with 4 GHz bandwidth, and is configured with two transmitting antennas and two receiving antennas, forming an L-shaped interferometric baseline for angle estimation. The mmWave sensor is positioned 0.9 m above the ground, and each volunteer walks back and forth within a 5 m range from the radar. During data collection, eleven volunteers sequentially completed walking with the four arm movements experienced. The experiment captured approximately 100 s of radar echo data for each volunteer, so a total of 11 × 4 × 100 s of radar raw data is acquired.

The collected radar raw data was processed following the flow chart of Figure 1. First, range and azimuth processing are conducted, followed by 3D-CFAR target detection to obtain the point-cloud data. Then multi-channel correlation processing is used to obtain the elevation and azimuth angular information used for extracting different limb motion data.

**Figure 8:**
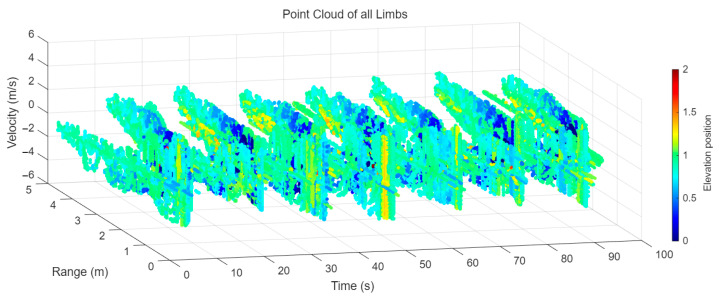
Point-cloud data from all limbs of a pedestrian.

Figure 8 displays the point cloud from all moving limbs of an study subject, where the color indicates elevation information, represented by the height relative to the ground. The point cloud can be classified into two main components, a light-blue component referring to heights above 0.9 m and a dark-blue component referring to heights below 0.9 m. Since the arms are usually positioned above the legs when walking, the light-blue portion likely corresponds to the arm movements, while the dark-blue portion likely represents leg movements. In the range dimension, the human target moves back and forth within a range of 5 m. In the velocity dimension, the average walking speed is around 1 m/s, representing the main body’s velocity. Due to the swinging motion of arms and the movements of legs are around the torso, their velocities oscillate around the main body speed too, resulting in a wide dynamic range of velocities centered at 1 m/s. It should be noted that in Figure 8, the positive/negative values of velocity represent the movement direction of the human target. The velocity is defined as negative when the target moves towards the sensor and positive when moving away from it. As shown in Figure 8, negative velocity values appear between 0 and 5 s while the target walks toward the radar. When the target turns around and moves away from the radar between 5 and 10 s, the velocity values become positive.

From Figure 8, it can be observed that the light-blue point cloud corresponding to arm movements occupies a significant portion of the overall point-cloud shape, directly influencing the extracted features. Unlike the legs, which provide support during walking, the arms are highly flexible and can exhibit various swinging patterns depending on social contexts. Consequently, the shape and features of the arm-related point cloud vary considerably.

In gait recognition applications, if new arm movement patterns (not present in the training data) appear, the resulting arm-induced point-cloud features may overshadow the original leg movement features, leading to degraded recognition performance. Since this is undesirable in practical applications, the point cloud is further processed to separate arm and leg data, extracting only the leg-related portion to reduce the impact of arm movement variations on gait recognition.

**Figure 9:**
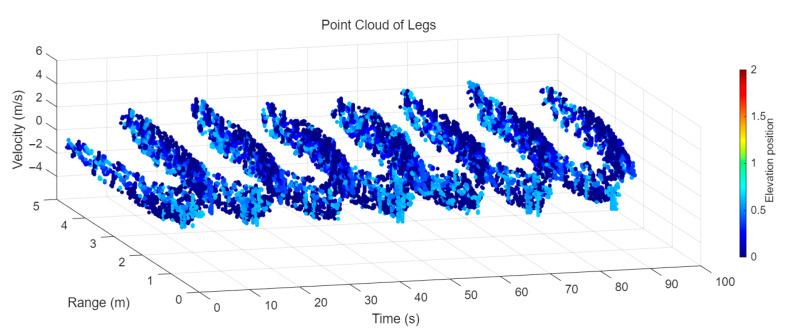
Point-loud data from the legs of a pedestrian.

Figure 9 presents the extracted leg-only motion data from Figure 8 that includes all limb data. This paper will utilize leg-only data to conduct human identity recognition based on gait features, as leg motion exhibits significantly stronger stability compared to data that includes arm movements. Under limited training samples, this approach avoids the impact of sudden arm motions on gait recognition.

As shown in Figure 8 and Figure 9, the point cloud constructed in the three-dimensional space of velocity, distance and time is used for subsequent gait recognition experiments, which can effectively represent the characteristics of human movement and is conducive to the feature extraction of point-cloud data for gait recognition [24]. A duration of 1.5 s is sufficient for most pedestrians to complete one gait cycle. Therefore, in the experiment, the point-cloud data is divided into 1.5 s samples to construct the gait recognition dataset.

### 4.2. Experimental Setup

The dataset contains the gait data from eleven individuals under four different arm scenarios, which are labeled as M1, M2, M3, and M4 (as shown in Table 1).

As mentioned earlier, the point-cloud data has been segmented into 1.5 s samples. Table 2 and Table 3 present the visualizations of some point-cloud samples, where the range information and time information have been normalized to the mean, so the value range is from −1 to 1. The color of the point cloud represents elevation angle, azimuth angle and signal intensity as red, green and blue channels.

Table 2 presents the point-cloud samples of the same person in four different arm movement states while walking. The second row of the table shows the point cloud of legs, and the third row shows the point cloud of all limbs. It can be intuitively seen that for the walking data of the four arm movements, the point-cloud samples of legs have higher feature consistency, while the point-cloud samples of all limbs show significant differences in the overall point cloud due to the changes in arm movements. Therefore, it is not conducive to gait-based identity recognition under different arm movement states.

Table 3 presents the point-cloud samples of different people under the same arm movement. Compared with the point-cloud samples of all limbs, the point-cloud samples of legs provide only the motion characteristics of the legs. Since the motion characteristics of the legs are relatively stable, it can be more beneficial for the model to extract gait features more efficiently.

Based on this dataset, three different experiments are conducted based on the training and testing configurations as outlined in Table 4.

In Experiment 1, all four arm movements are included in both training and testing. In addition, gait recognition experiments with different training sample sizes are set up; 70%, 45% and 30% of the total samples of are used respectively in Exp. 1-0, Exp. 1-1 and Exp. 1-2 for training, while the remaining 30% of the total samples are reserved for testing.

In Experiments 2 and 3, gait recognition performances are tested with only a subset of arm movements used for training, while testing is performed on data containing previously unseen arm movements (new arm movements not included in training). This setup corresponds to the challenges of practical applications where it is difficult to collect and train on all possible arm movements due to the high flexibility of human arm motion. These experiments aim to evaluate the robustness of gait recognition models when encountering new arm movement patterns have not been trained, ensuring reliable performance in real-world applications where arm behavior may vary unpredictably.

To validate the effectiveness of the proposed gait recognition method that utilizes only leg motion data, comparative experiments are conducted between the approach based solely on leg motion data and the existing method based on all limb motion data. As shown in Table 5, the proposed method using only leg point-cloud data is named the Leg Method, while the traditional approach employing full-body point-cloud data is referred to as the Total Method.

As shown in Figure 6, the experiments employ the point-cloud deep learning model PointNet++, and utilize the Adam optimizer with the learning rate set to be 0.001. The network model was implemented and trained using the PyTorch 1.8 framework on an NVIDIA Titan Xp GPU (with 12 GB VRAM and 3840 CUDA cores).

The architecture of PointNet++ is presented in Table 6, where N represents the number of core points, and S denotes the number of neighboring points for each core point. The model employs multi-scale grouping and set abstraction (SA) layers to hierarchically extract point-cloud features, followed by fully connected (FC) layers for classification.

### 4.3. Experimental Results

#### 4.3.1. Experiment 1: All Four Arm Movements in Training Set

The training set of Experiment 1 is formed of the gait data with four arm movements included, and Exp. 1-0 uses 70% of the total samples as the training set. To evaluate the effect of varying training data sizes, Exp. 1-1 uses 45% of the total samples for training, while Exp. 1-2 uses 30%. Exp. 1-1 and Exp. 1-2 use the same test set as Exp. 1-0, which is the other 30% of data samples after 70% of them are selected as the training data.

Table 7 presents the gait recognition results under different training sample sizes. It can be observed that among the three experiments, Exp. 1-0 achieves the highest gait recognition accuracy regardless of whether the Leg Method or the Total Method was used. The gait recognition accuracy decreases as the training sample size decreases. It should be noted that the gait recognition accuracy was evaluated using a held-out test set. Prior to evaluation, the test set was rigorously isolated during training to avoid data leakage. Recognition performance was quantified as the percentage of correctly classified point-cloud samples relative to the total test samples.

Let us compare the results listed in Table 7 in detail. For the Leg Method, the accuracy results of Exp. 1-1 and Exp. 1-2 drop about 4.3% and 8.6%, respectively, compared to that of Exp. 1-0. As for the Total Method, the accuracies drop about 5.0% and 13%, respectively. It is clear that the Leg Method exhibited a smaller decline in gait recognition accuracy compared to the Total Method as the training sample size decreased. This is because the Total Method incorporates the diversity of arm movements, making the point cloud include not only leg motion characteristics but also various arm motion characteristics. When the training samples are reduced, the model’s ability to extract gait features becomes more susceptible to the interference from arm motion features, leading to a more significant drop in gait recognition accuracy.

**Figure 10:**
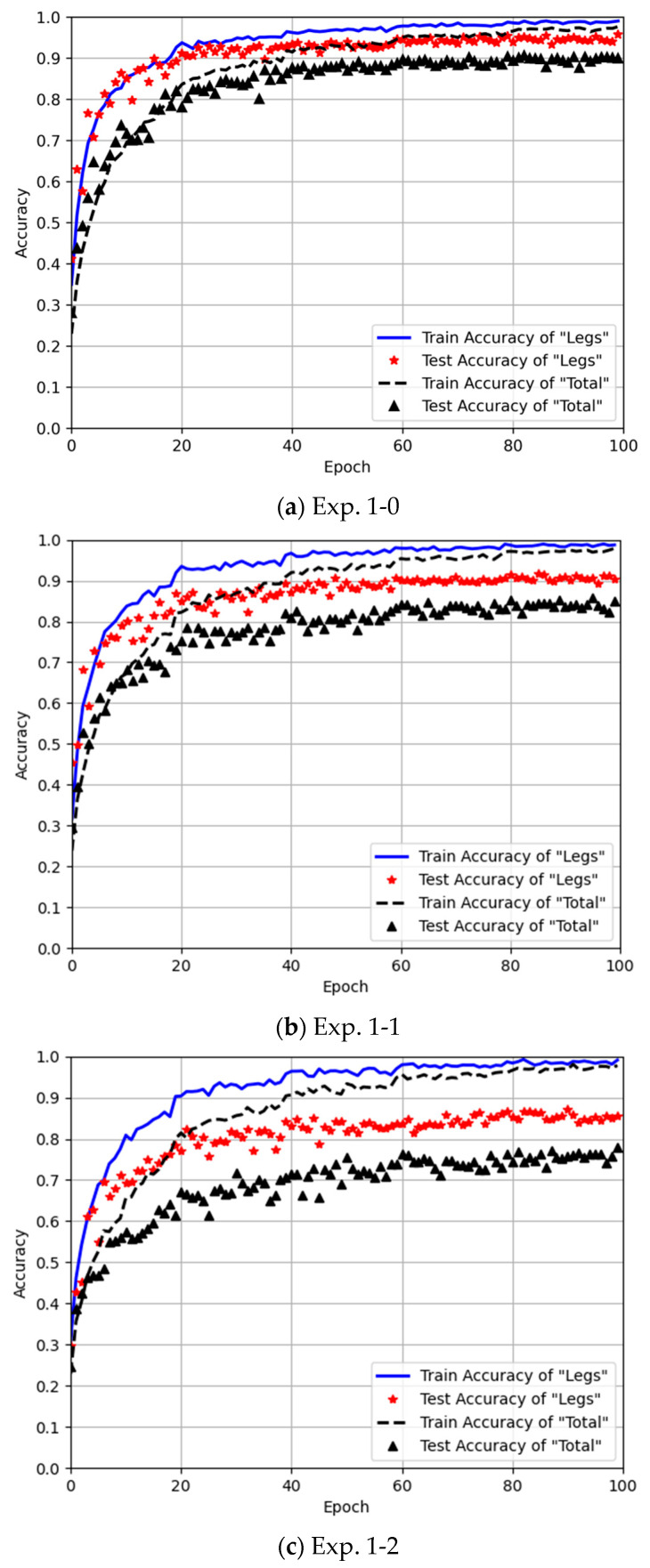
Training curves of the Leg Method and the Total Method for Experiment 1.

Figure 10 shows the training curves of Experiment 1 using the Leg Method and the Total Method under different training sample sizes. The blue solid line represents the training accuracy of the Leg Method, while the red asterisks denote its testing accuracy. The black dashed line indicates the training accuracy of the Total Method, and the black triangles represent its testing accuracy.

From the testing accuracy results in Figure 10a, it can be observed that after 100 training epochs, the proposed Leg Method achieves a testing accuracy of 95.8%, outperforming the existing Total Method (90.8%) by a significant margin. Additionally, at epoch 20, the Leg Method already reaches 90% accuracy, whereas the Total Method only attains 80%.

When the training samples are reduced, as shown in Figure 10b,c, the Leg Method maintains a minimum accuracy of above 80% at epoch 20, while the Total Method struggles to exceed 80% even at its peak. Across all sample sizes in Experiment 1, the proposed Leg Method consistently achieves at least 5% higher accuracy than the Total Method. Moreover, as the training samples decrease, the Total Method exhibits a more pronounced decline in gait recognition accuracy and is more prone to overfitting due to insufficient training data.

This indicates that the Leg Method can better learn the gait features of human subjects in training, leading to faster convergence and more robust performance even with limited training samples.

**Figure 11:**
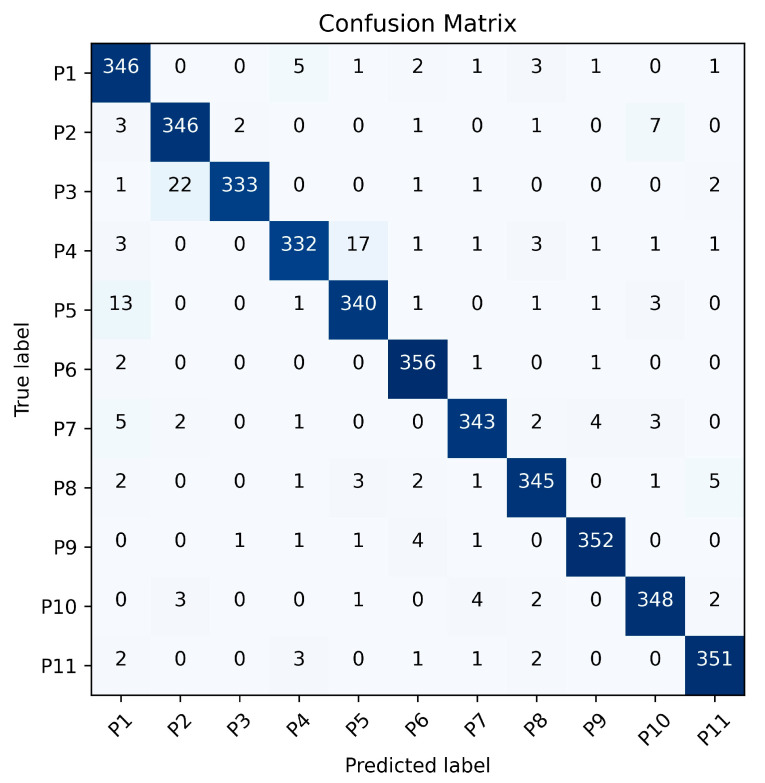
Confusion matrix of the Leg Method for Exp. 1-0.

**Figure 12:**
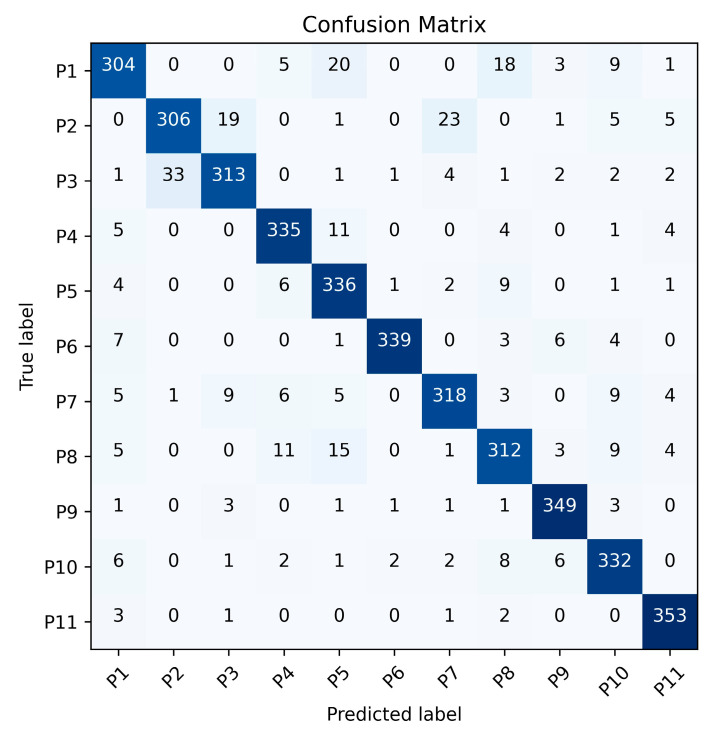
Confusion matrix of the Total Method for Exp. 1-0.

To further analyze the recognition results of the Leg Method and the Total Method, Figure 11 and Figure 12 present the gait recognition confusion matrices for Exp. 1_0 using the Leg Method and the Total Method respectively. These matrices offer a visual representation of the classification performance for each of the 11 subjects under the two methods. By examining these matrices, we can not only observe the misclassification patterns but also calculate the recognition precision, recall and F1-score for each person and the overall accuracy of the two methods, which helps in comprehensively evaluating the effectiveness of the Leg Method and the Total Method in gait recognition.

The results represented in Table 8 demonstrate that the proposed Leg Method consistently outperforms the Total Method across all participants (P1–P11), achieving superior precision (91.8–99.1%), recall (92.2–98.9%), and F1-scores (93.8–97.8%) compared to the Total Method’s precision (85.7–98.6%), recall (84.4–98.1%), and F1-scores (86.6–96.3%). The Leg Method exhibits exceptional robustness, with all metrics exceeding 90%, confirming its reliability in minimizing false positives while maintaining high detection accuracy. Although the Total Method shows competitive performance in some cases (e.g., P6 and P11), its greater variability highlights the Leg Method’s overall superiority in consistency and effectiveness.

#### 4.3.2. Experiment 2: Three Arm Movements in Training Set

We aimed to investigate whether the model still can accurately identify the target individual’s identity or not when gait data with one or more arm movements are absent from training but appear in testing. Experiment 2, as shown in Table 4, uses the gait data containing three arm movements to train the model and the remaining one arm movement’s gait data for testing. As for Exp. 2_1, it uses M2, M3, and M4 for training while using M1 for testing. Similarly, Exp. 2_2 uses M1, M3, and M4 for training while using M2 for testing; Exp. 2_3 uses M1, M2, and M4 for training while using M3 for testing; and Exp. 2_4 uses M1, M2, and M3 for training while using M4 for testing.

The recognition results of Experiment 2 are listed in Table 9, showing that even when trained by the gait data of three arm movements, the Leg Method can still achieve the minimum recognition accuracy of 89.4% and the maximum of 96.9%. In contrast, the Total Method achieves a minimum of 77.6% and a maximum of 93.2%.

From Table 9, two conclusions can be drawn:

1. Different arm movements affect the model training effect differently, thereby influencing the recognition accuracy in case of new arm movements appearing.

- The results of Exp. 2_2 show that, when using M1, M3, and M4 as the training set and M2 as the test set, the Leg Method achieves 93.9% recognition accuracy, while the Total Method reaches only 77.6%. This is because the swinging characteristics of M2 are significantly different from those of M1, M3, and M4, but it is absent from training. As for the Total Method, due to all limb movements being included, the gait recognition is severely affected by drastic changes in arm movements. In contrast, with the Leg Method, due to only the leg movement data being utilized, the impact of arm movement variations can be avoided.

- The results of Exp. 2_3 show that, when M1, M2, and M4 are used for training with M3 for testing, both methods can achieve high recognition accuracy. This is because the absent M3 involves single-arm swinging, sharing the similar characteristics of M1 with two arms swinging, allowing the model to leverage the learned features of M1 when training. Due to the similarity between M3 and M1, the recognition results of Exp. 2_3 are comparable to those of Exp. 1_0, even being slightly higher, approximately 1% for the Leg Method and 3% for the Total Method. This can be attributed to the Leg Method’s reliance solely on leg data, so the influence of arm movement variations on gait recognition can thus be reduced. Compared to the case of Exp. 2_3, Exp. 1_0 introduces one more arm movement, whose feature may influence the model’s extraction of gait features. Therefore, with fixed training samples, even when one arm movement type is added, the gait recognition accuracy can be reduced. More types of arm movements should require a larger number of training samples to achieve better recognition accuracy. Since this experiment involves a limited number of arm movements, further experiments are needed to validate this hypothesis more reliably.

2. The proposed Leg Method maintains high gait recognition accuracy under different scenarios. Across all four experimental groups in Experiment 2, Leg Method consistently outperforms the Total Method in recognition accuracy, particularly when encountering arm movements significantly different from those in training; e.g., in Exp. 2_2, stable and high gait recognition accuracy performance is demonstrated.

**Figure 13:**
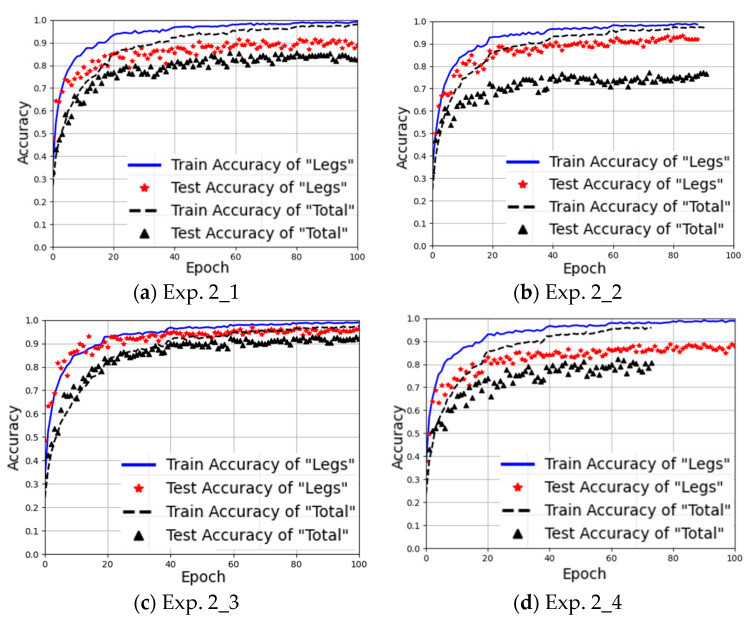
Training curves of the Leg Method and the Total method for Experiment 2.

Figure 13 presents the training curves of the four experimental groups in Experiment 2. It can be observed that the Leg Method not only achieves higher recognition accuracy but also demonstrates faster model convergence speed. This further confirms that the proposed Leg Method facilitates more effective extraction of gait features by the model, thereby better maintaining gait recognition accuracy even when the arm movements change differently.

#### 4.3.3. Experiment 3: Two Arm Movements in Training Set

Experiment 3 employes gait data with two arm movements included for model training while using the gait data with the remaining two arm movements as test sets. Let us explain the meanings of different setups by taking Exp. 3_12-3, Exp. 3_12-4, and Exp. 3_12-34 as examples. Exp. 3_12-3 denotes the case with M1 and M2 used for training and M3 for testing; Exp. 3_12-4 denotes the case with M1 and M2 used for training and M4 for testing; and Exp. 3_12-34 denotes the case with M1 and M2 used for training and M3 and M4 both used for testing.

Table 10 presents the results of Experiment 3, from which the recognition performances under different scenarios can be analyzed.

Compared to Experiment 1 and Experiment 2, Experiment 3 employs only two arm movements for training; i.e., not only fewer types of arm movements are involved but also the training samples are reduced as well. Consequently, model training in Experiment 3 faces a greater challenge. As shown in Table 10, the gait recognition accuracies in Experiment 3 are relatively lower than those in Experiment 1 (as shown in Figure 10) and Experiment 2 (as listed in Table 9), and the reason is primarily the reduced training variety and sample size.

Table 10 clearly shows that the Leg Method consistently outperforms the Total Method in recognition accuracy, and the largest accuracy difference of 22.90% occurs in Exp. 3_34-2, while the smallest difference of 2.60% appears in Exp. 3_14-3. The reason is analyzed as follows.

Exp. 3_14-3 uses M1 and M4 for training and M3 for testing. Since the arm movement characteristics of M3 resemble M1, the model had already learned similar features during training. As a result, both the Total Method and Leg Method achieve over 90% recognition accuracy when tested on M3. The configuration of Exp. 3_14-3 is similar to that of Exp. 2_3. However, due to the larger training sample size and variety in Exp. 2_3, the recognition accuracy slightly outperforms Exp. 3_14-3, achieving 96.9% and 93.2% with the Leg Method and Total Method, respectively, compared to 93.0% and 90.4% for Exp. 3_14-3.

Exp. 3_34-2 uses M3 and M4 for training, while M2 is used for testing. Since the training set consisted of relatively stable or motionless arm movements, the model failed to learn the highly dynamic arm swing patterns of M2. Consequently, the Total Method suffer a significant drop in accuracy as low as 65.9% due to the interference from M2’s arm movements, whereas the Leg Method maintained a high accuracy of 88.8%, because it is immune to the influence of arm movement. All the results demonstrate that under challenging conditions where test and training samples differ substantially, the performance of the Total Method degrades sharply, while the Leg Method remains robust.

**Figure 14:**
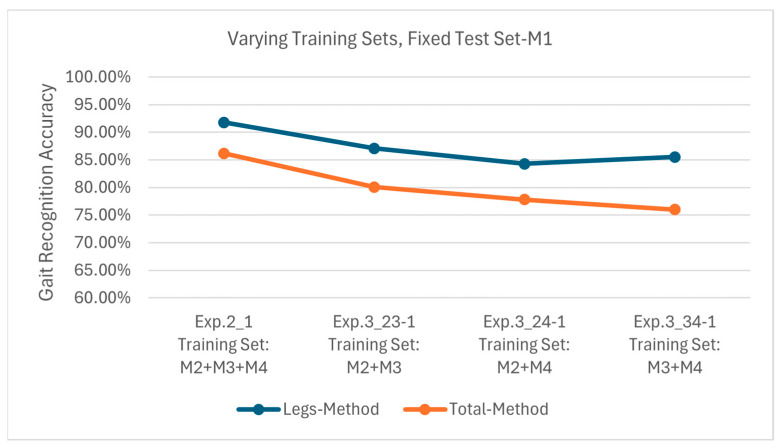
Recognition accuracies using test set M1 on models obtained from different training sets.

Figure 14 presents the results of Exp. 2_1, 3_23-1, 3_24-1, and 3_34-1, all of which use M1 as the test set. The Leg Method achieves recognition accuracies of 91.8%, 87.1%, 84.3%, and 85.5%, respectively, while they are 86.2%, 80.1%, 77.8%, and 76.0%, respectively, for the Total Method. Since M1, M2, and M3 all involve arm movements, and the training sets includes M2 or M3, the model has already learned similar motion features in training. Thus, stable recognition accuracy is obtained across different training configurations. Notably, the minimum recognition accuracy of the Leg Method can also reach 84.3%, while the maximum recognition accuracy of the Total Method is only 86.2%, further validating the superiority of the Legs Method in handling arm movement variations.

**Figure 15:**
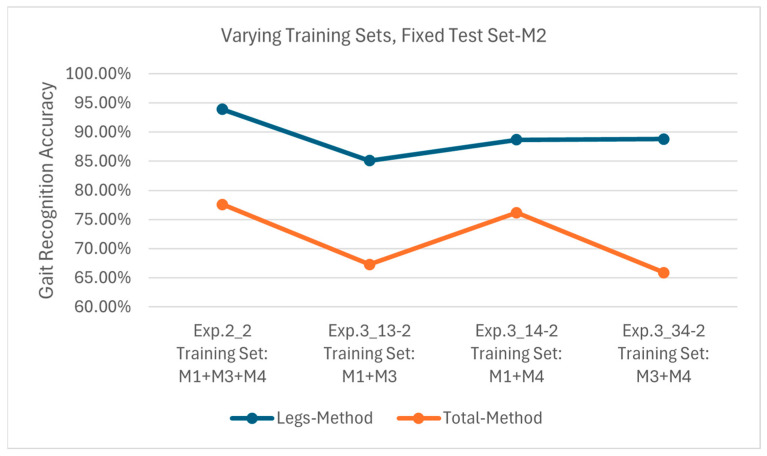
Recognition accuracies using test set M2 on models obtained from different training sets.

Figure 15 presents the results of Exp. 2_2, 3_13-2, 3_14-2 and 3_34-2, all using M2 as the test set. The Leg Method achieves recognition accuracies of 93.9%, 85.1%, 88.7% and 88.8%, respectively, while they are 77.6%, 65.9%, 76.2% and 67.3%, respectively, for the Total Method. Compared to M1 and M3, M2’s arm movements exhibit greater diversity and complexity. Consequently, when M2 is used for testing with the Total Method, the model suffers from significant accuracy degradation due to arm movement variations; thus, the maximum and minimum accuracies are only about 77.6% and 65.9%, respectively. In contrast, the proposed Leg Method can effectively mitigate the impact of different arm movements and maintain a recognition accuracy above 85%.

**Figure 16:**
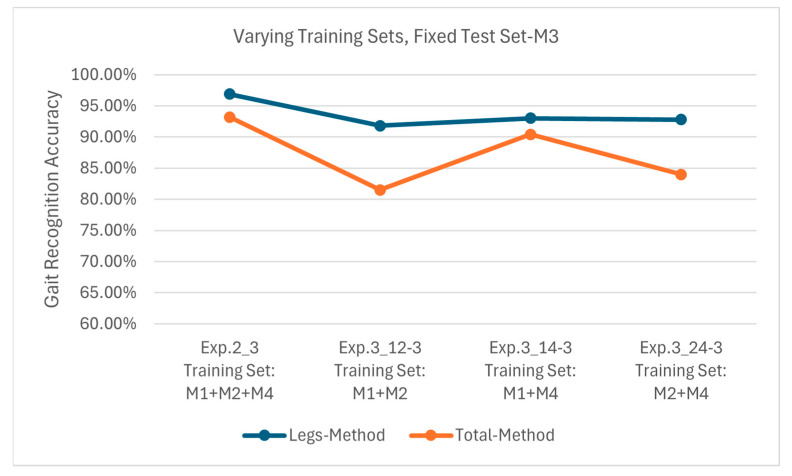
Recognition accuracies using test set M3 on models obtained from different training sets.

Figure 16 shows the results of Exp. 2_3, 3_12-3, 3_14-3 and 3_24-3, all employing M3 as the test set. The Leg Method achieves 96.9%, 91.8%, 93.0% and 92.8% accuracies, respectively, while the Total Method achieves the accuracies of 93.2%, 81.5%, 90.4% and 84.0%, respectively. As previously illustrated for Exp. 2_3, M3’s single-arm swinging shares similarities with M1’s double-arm swinging, and M2’s random swinging also contains some arm movement features. This enables both methods to achieve relatively high accuracy when testing with M3, and at the same time, the Leg Method demonstrates superior stability in maintaining high accuracy.

**Figure 17:**
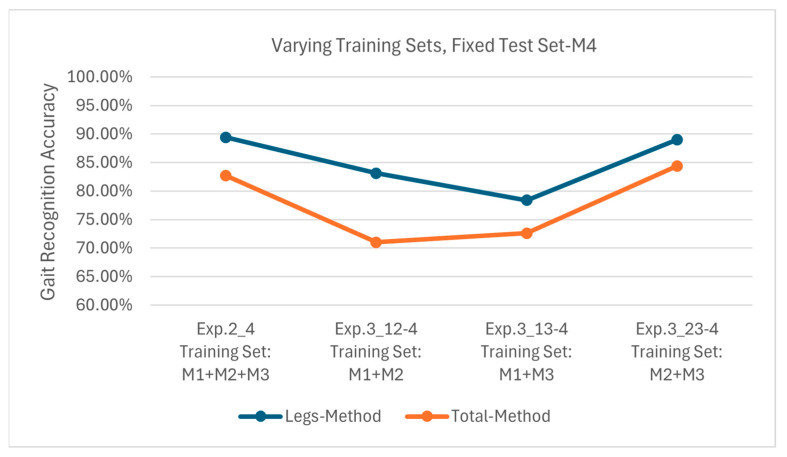
Recognition accuracies using test set M4 on models obtained from different training sets.

Figure 17 displays the results of Exp. 2_4, 3_12-4, 3_13-4 and 3_23-4, all using M4 as the test set. The Leg Method attained 89.4%, 83.1%, 78.4% and 89.0% accuracies, respectively, and the corresponding accuracies of the Total Method are 82.7%, 71.0%, 72.6% and 84.4%, respectively. Since M4 involves no arm movement while M1–M3 all contain arm motions, there exists significant discrepancy between the training and testing sets regarding arm movement states. This leads to notably lower accuracies for the Total Method when testing with M4; e.g., the maximum is 84.4% and the minimum is 71.0%. Despite the pronounced arm movement differences, the Leg Method still outperforms the Total Method with accuracies ranging from 78.4% to 89.4%.

The above three experiments show that when the model is trained with different sets, the recognition performances on the same test set can be quite different, highlighting the critical importance of training sample diversity for gait recognition. In practical applications, dataset construction is costly, making the trade-off between the training costs and the recognition accuracy a crucial consideration. Compared with the existing Total Method, the proposed Leg Method can significantly improve the recognition accuracy without increasing training costs while maintaining high recognition accuracy, even in cases that the testing gait data contains arm movements that are absent from training. All the results demonstrate that the Leg Method can be a substantially potential approach for simultaneously reducing training costs and enhancing recognition accuracy.

## 5. Conclusions

Arm movements of various kinds usually accompany walking. This paper carried out experiments studying the influence of different gestures on gait-based identity recognition. The experimental results demonstrate that the proposed method using lower limb data has the potential to address the trade-off between recognition accuracy and training costs. Although increasing the diversity of arm movements and expanding the training dataset can enhance gait recognition performance, such strategies will inevitably escalate the computational and data collection burdens. The proposed method leverages extracted lower limb movement features for gait-based human identification; it not only can achieve high accuracy and robust stability but also can substantially reduce the model training overhead, and this advantage is key to better handle gait data with different arm motion patterns. We plan to conduct further in-depth experiments and better utilize different limbs’ (including arms) motion data for downstream applications like gesture recognition, aiming to achieve a more comprehensive recognition application.

## Figures and Tables

**Table 1:** Four arm motions when walking.

Naming	Description of Arm Motion	Simulated Walking Scenario
M1	With the gait cycle, both arms swing periodically	A typical walking state
M2	Both arms swing randomly in a non-periodic manner	Making gestures during communication and interaction
M3	One hand holds an object, and the other arm swings naturally	Looking at a mobile phone or holding objects with one hand
M4	Both hands hold objects, arms fixed, no arm swing	Looking at a mobile phone and typing or holding objects with both hands

**Table 2:** Point-cloud samples of the same person in different arm movement states while walking.

	M1	M2	M3	M4
Point-cloud sample of legs	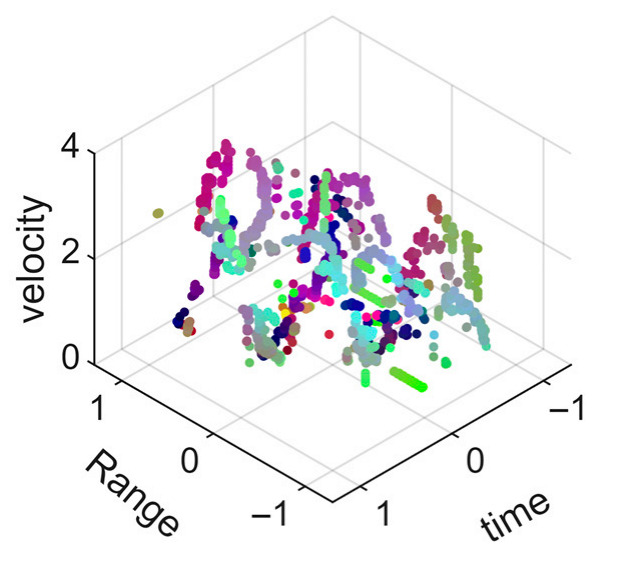	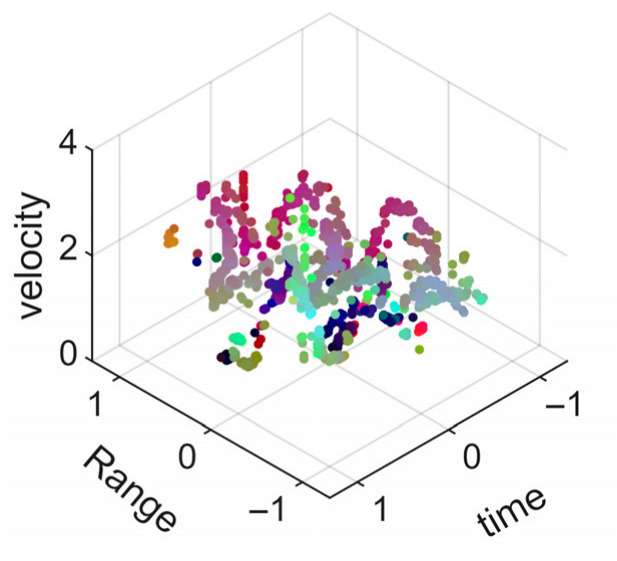	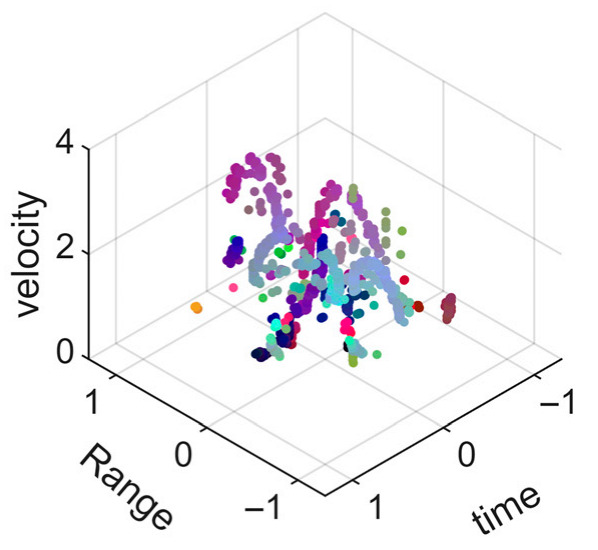	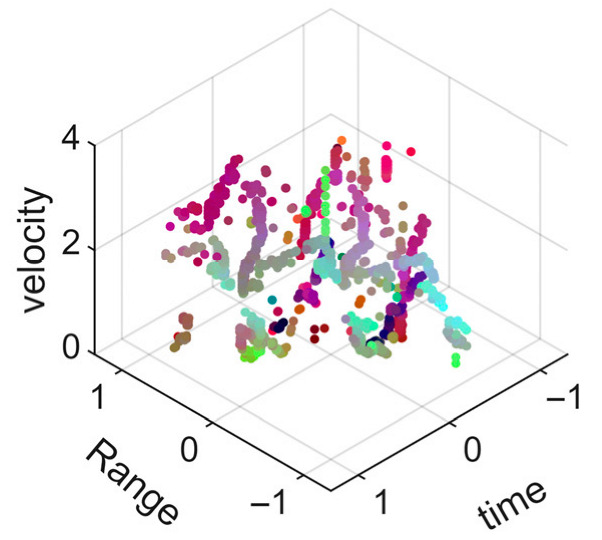
Point-cloud sample of all limbs	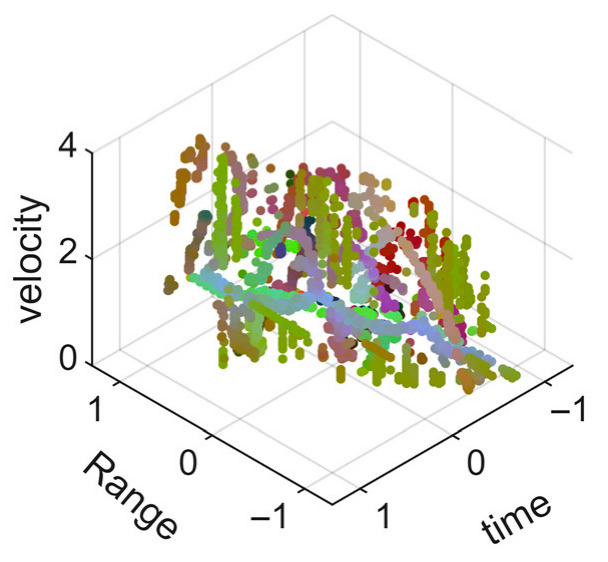	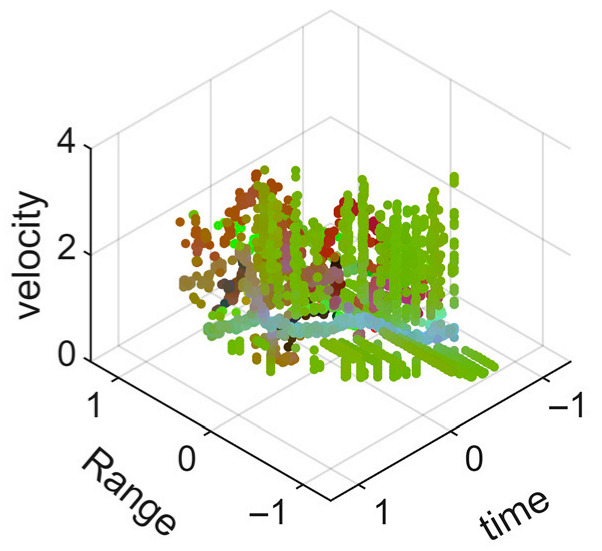	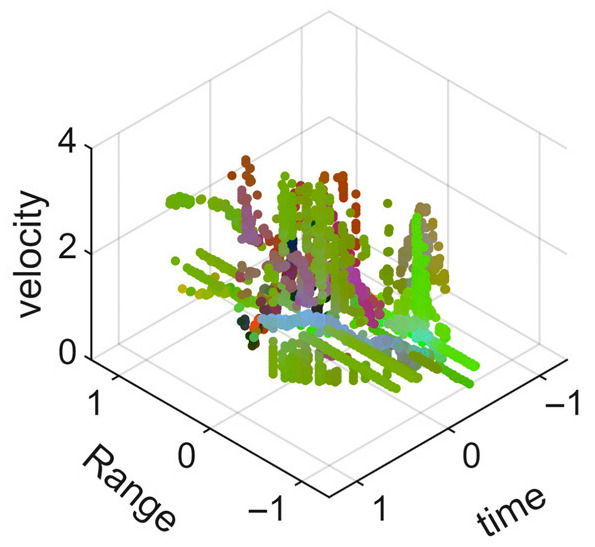	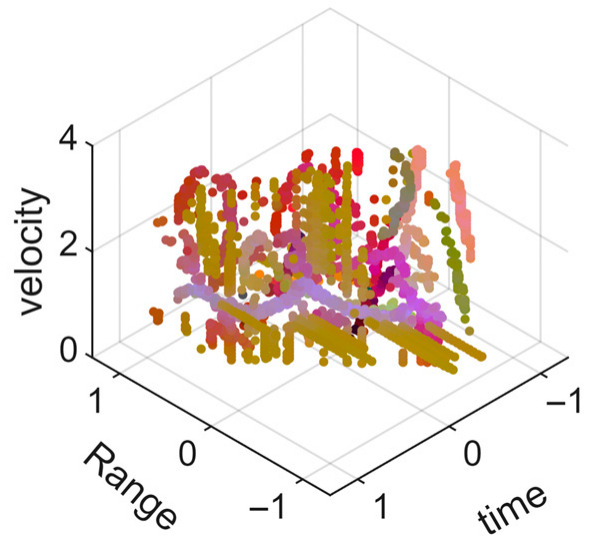

**Table 3:** Point-cloud samples of different persons in same arm movement state while walking.

	Person 1	Person 2	Person 3	Person 4
Point-cloud sample of legs	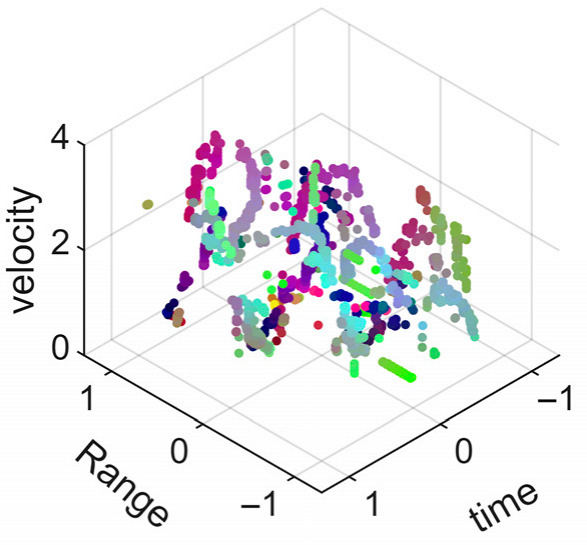	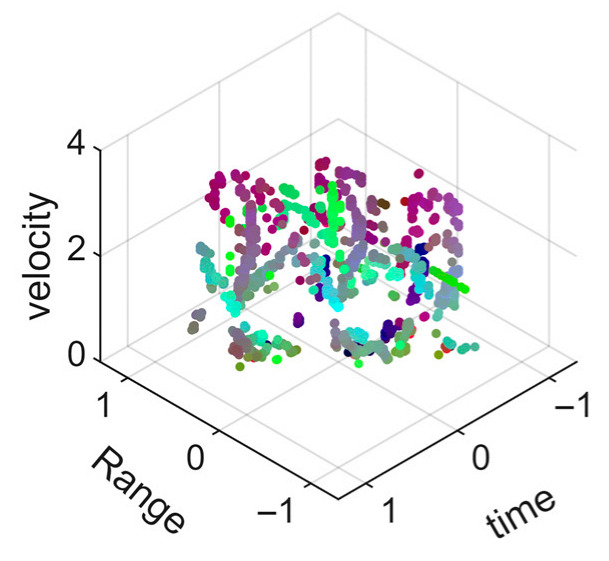	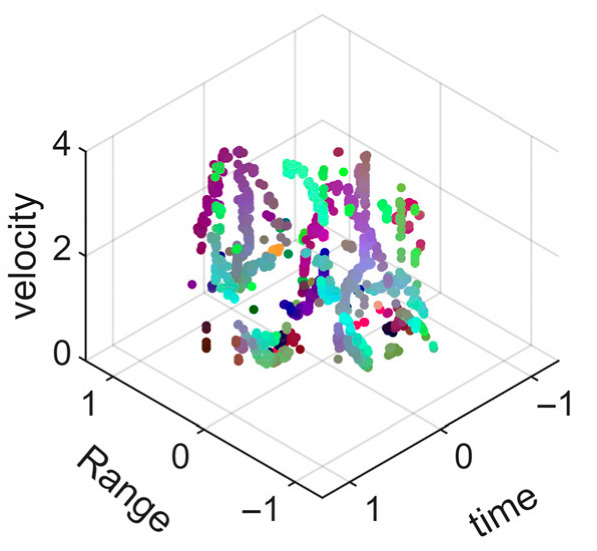	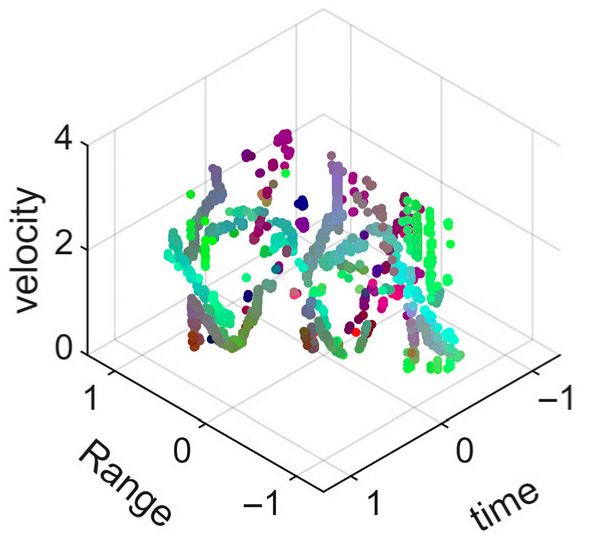
Point-cloud sample of all limbs	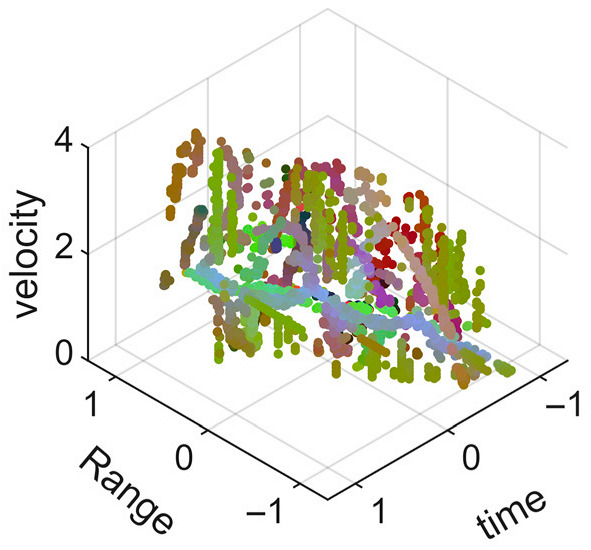	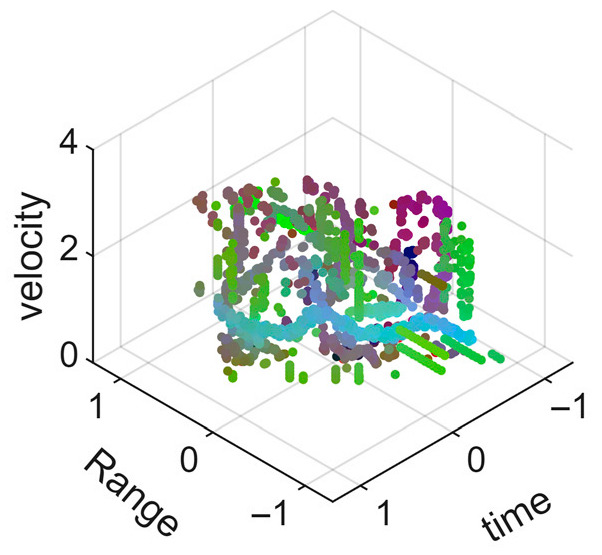	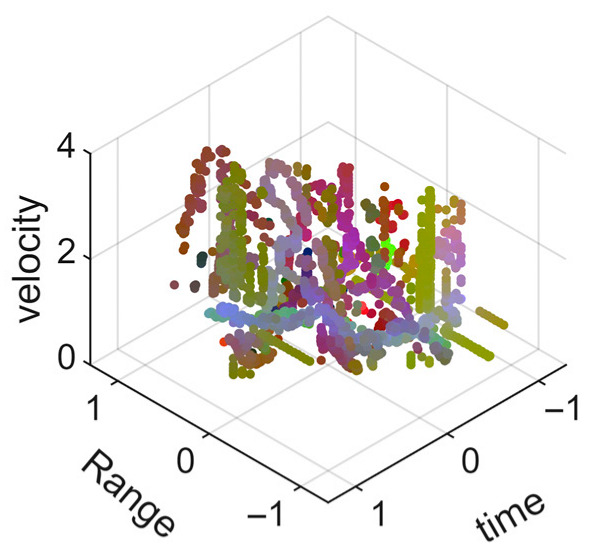	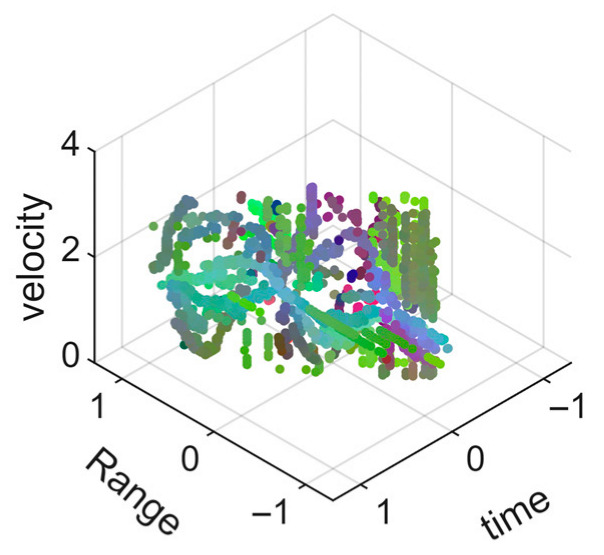

**Table 4:** Experiment training and test sample configuration.

	Training Set Composition	Test Set Composition	Experiment Naming	Size of Training Sample
Experiment 1	(M1 + M2 + M3 + M4) × 70%	(M1 + M2 + M3 + M4) × 30%	Exp. 1-0	8443
	(M1 + M2 + M3 + M4) × 45%	(M1 + M2 + M3 + M4) × 30%	Exp. 1-1	5803
	(M1 + M2 + M3 + M4) × 30%	(M1 + M2 + M3 + M4) × 30%	Exp. 1-2	3603
Experiment 2	(M2 + M3 + M4) × 100%	(M1) × 100%	Exp. 2_1	9139
	(M1 + M3 + M4) × 100%	(M2) × 100%	Exp. 2_2	9443
	(M1 + M2 + M4) × 100%	(M3) × 100%	Exp. 2_3	9228
	(M1 + M2 + M3) × 100%	(M4) × 100%	Exp. 2_4	9399
Experiment 3	(M1 + M2) × 100%	(M3) × 100%	Exp. 3_12-3	6224
	(M1 + M2) × 100%	(M4) × 100%	Exp. 3_12-4	6224
	(M1 + M2) × 100%	(M3 + M4) × 100%	Exp. 3_12-34	6224
	(M1 + M3) × 100%	(M2) × 100%	Exp. 3_13-2	6439
	(M1 + M3) × 100%	(M4) × 100%	Exp. 3_13-4	6439
	(M1 + M3) × 100%	(M2 + M4) × 100%	Exp. 3_13-24	6439
	(M1 + M4) × 100%	(M2) × 100%	Exp. 3_14-2	6268
	(M1 + M4) × 100%	(M3) × 100%	Exp. 3_14-3	6268
	(M1 + M4) × 100%	(M2 + M3) × 100%	Exp. 3_14-23	6268
	(M2 + M3) × 100%	(M1) × 100%	Exp. 3_23-1	6135
	(M2 + M3) × 100%	(M4) × 100%	Exp. 3_23-4	6135
	(M2 + M3) × 100%	(M1 + M4) × 100%	Exp. 3_23-14	6135
	(M2 + M4) × 100%	(M1) × 100%	Exp. 3_24-1	5964
	(M2 + M4) × 100%	(M3) × 100%	Exp. 3_24-3	5964
	(M2 + M4) × 100%	(M1 + M3) × 100%	Exp. 3_24-13	5964
	(M3 + M4) × 100%	(M1) × 100%	Exp. 3_34-1	6179
	(M3 + M4) × 100%	(M2) × 100%	Exp. 3_34-2	6179
	(M3 + M4) × 100%	(M1 + M2) × 100%	Exp. 3_34-12	6179

**Table 5:** Abbreviations of gait recognition methods based on point-cloud data.

Method Name	Description
Leg Method	The proposed method uses leg data only
Total Method	The existing method uses all limb data

**Table 6:** Parameter configuration of PointNet++ architecture modules.

Module	Parameters	Description
SA1	N = 512, S = [16, 32, 128], MLP = [[32, 32, 64], [64, 64, 128], [64, 96, 128]]	Multi-scale local feature extraction
SA2	N = 128, S = [32, 64, 128], MLP = [[64, 64, 128], [128, 128, 256], [128, 128, 256]]	Hierarchical feature aggregation
SA3	MLP = [256, 512, 1024]	Global feature extraction
FC Layers	MLP = [1024, 512, 256, 11]	Classification

**Table 7:** Recognition accuracies using different training samples for Experiment 1.

	Leg Method	Total Method	∆
Exp. 1-0	95.8%	90.8%	5.0%
Exp. 1-1	91.5%	85.8%	5.7%
Exp. 1-2	87.2%	77.8%	9.4%

**Table 8:** The precision, recall and F1-score for Exp. 1-0.

	Precision		Recall		F1-Score	
	Leg Method	Total Method	Leg Method	Total Method	Leg Method	Total Method
P1	91.8%	89.2%	96.1%	84.4%	93.9%	86.7%
P2	92.8%	90.0%	95.6%	85.0%	94.2%	87.4%
P3	99.1%	90.5%	92.5%	86.9%	95.7%	88.7%
P4	96.5%	91.8%	92.2%	93.1%	94.3%	92.4%
P5	93.2%	85.7%	94.4%	93.3%	93.8%	89.4%
P6	96.5%	98.6%	98.9%	94.2%	97.7%	96.3%
P7	96.9%	90.3%	95.3%	88.3%	96.1%	89.3%
P8	96.1%	86.4%	95.8%	86.7%	96.0%	86.6%
P9	97.8%	94.3%	97.8%	96.9%	97.8%	95.6%
P10	95.9%	88.5%	96.7%	92.2%	96.3%	90.3%
P11	97.0%	94.4%	97.5%	98.1%	97.2%	96.2%

**Table 9:** Recognition accuracies of Leg and Total Methods for Experiment 2.

	Leg Method	Total Method	∆
Exp. 2_1	91.8%	86.2%	5.6%
Exp. 2_2	93.9%	77.6%	16.3%
Exp. 2_3	96.9%	93.2%	3.7%
Exp. 2_4	89.4%	82.7%	6.7%

**Table 10:** Recognition accuracies of Leg and Total Methods for Experiment 3.

	Leg Method	Total Method	∆
Exp. 3_12-3	91.8%	81.5%	10.30%
Exp. 3_12-4	83.1%	71.0%	12.10%
Exp. 3_12-34	87.5%	76.4%	11.10%
Exp. 3_13-2	85.1%	67.3%	17.80%
Exp. 3_13-4	78.4%	72.6%	5.80%
Exp. 3_13-24	81.8%	70.3%	11.50%
Exp. 3_14-2	88.7%	76.2%	12.50%
Exp. 3_14-3	93.0%	90.4%	2.60%
Exp. 3_14-23	91.1%	82.6%	8.50%
Exp. 3_23-1	87.1%	80.1%	7.00%
Exp. 3_23-4	89.0%	84.4%	4.60%
Exp. 3_23-14	88.2%	82.5%	5.70%
Exp. 3_24-1	84.3%	77.8%	6.50%
Exp. 3_24-3	92.8%	84.0%	8.80%
Exp. 3_24-13	88.7%	80.9%	7.80%
Exp. 3_34-1	85.5%	76.0%	9.50%
Exp. 3_34-2	88.8%	65.9%	22.90%
Exp. 3_34-12	86.9%	69.4%	17.50%

## Data Availability

The datasets presented in this article are not readily available. Requests to access the datasets should be directed to hexianxian@chinamobile.com.
